# Complete Removal of the Stomach

**Published:** 1898-01-08

**Authors:** 


### COMPLETE REMOVAL OF THE STOMACH.

A most interesting case of entire resection of the stomach, with œsophago-enterostomy, is recorded in the New York *Medical Record.* The patient was a woman, 56 years of age, who was suffering from cancer of the stomach. The points of interest are that the entire stomach was removed, the œsophagus being cut at the one end and the duodenum at the other, and that, it being found impossible to attach the two cut ends to each other, the divided œsophagus was made to open into a coil of jejunum situated about 15 inches from the duodenum, the cut end of the duodenum having been closed by invagination. The operation was performed by Dr. Carl Schlatter, of Zurich, on September 6th, 1897, and when seen on December 9th, save for some degree of emaciation, a noticeably dry skin, and her abdominal cicatrix, the patient offered no apparent departure from ordinary health.

